# Future expectation levels of adolescents during the COVID-19 pandemic

**DOI:** 10.3389/fpubh.2023.1199280

**Published:** 2023-11-30

**Authors:** Osman Küçükkelepçe, Mehmet Emin Parlak, Erdoğan Öz, Yaşar Kapıcı, Dilek Ener

**Affiliations:** ^1^Adıyaman Provincial Health Directorate, Adiyaman, Türkiye; ^2^Adıyaman Training and Research Hospital, Adiyaman, Türkiye; ^3^Kahta State Hospital, Adiyaman, Türkiye; ^4^Kırıkkale Provincial Health Directorate, Kırıkkale, Türkiye

**Keywords:** future expectation, adolescent, COVID-19, pandemic, high school

## Abstract

**Introduction:**

This study was aimed at examining the future expectations of high school students during the COVID-19 pandemic.

**Methods:**

A future expectation questionnaire was administered to 412 high school students, and the change in their expectations for the future during the COVID-19 pandemic period was questioned in this questionnaire.

**Results:**

Scale sub-scores and total scores of those who were infected with COVID-19 were significantly lower than those who were not (*p* < 0.001). The scale sub-scores and total scores of those whose relatives were infected with COVID-19 were significantly lower than those who were not (*p* < 0.05). The scale sub-scores and total scores of those who think that the pandemic will always continue, those who think that their education is disrupted due to the pandemic, those who think that the pandemic affects their choice of profession, and those who express that they look to the future with more hope than before the pandemic were found to be significantly lower (*p* < 0.05).

**Discussion:**

The future expectation is a more sensitive concept among adolescents than adults. During and after the pandemic, activities should be organized by considering the sensitivity of young people about their future expectations, and families and society should be made aware of this issue.

## 1 Introduction

COVID-19 was declared a pandemic by the WHO on 11 March 2020 as a result of the global spread of cases after the first case was seen in China on 17 November 2019. The first definitive case in Turkey occurred on the same day as the declaration of COVID-19 as an epidemic (1). With the spread of cases, it was announced by the Ministry of Health that the epidemic spread to the whole country on 1 April 2020. Adolescents appear as the most vulnerable group in the closures that started and continued during the pandemic, as they could not complete their psychosocial development (2). Adolescence is the period in which individuals prepare for their independent lives in adulthood and imagine their future health and social lives (3). Social isolation was at the forefront of the measures taken by the countries against the pandemic. This, in turn, caused changes in adolescents’ psychological states by causing isolation from school, friends, and teachers (4). Many studies in the literature have supported this situation (5–7).

According to the Turkish Language Association (TLA), expectation can be defined as what is expected to happen or as a person’s foresight about what is expected from him. Foresight can be influenced by an individual’s experiences. An individual with good past experiences will be more motivated and optimistic in line with his goals with the effect of optimistic thinking, and this will contribute positively to the future success of the individual (8). In this case, adolescents remain at a disadvantage compared to adults. Because adults have achieved a certain standard in their work, academic, and family lives, while adolescents plan their future lives, they have to adapt to globally developing technology and changing understandings. Future expectation can be defined as the desire or hope that a person’s future life will be more satisfying. This definition can be associated with the questions, “Will I have a happy life in the future?” “Will my expectations for the future come true?” “What kind of person will I be in the future?” “What will the world be like in the future?” (3). It is possible that adolescents with high future expectations are ahead in features such as social adaptation, the ability to make their own decisions, and gaining positive values (8). In another study conducted by Parsons et al., it was observed that future expectations and performance were correlated (9).

Adolescents with high future expectations will stay away from various harmful behaviors and addictions. For this, a climate that will raise the future expectations of adolescents in schools and social life should be created. In our study, the future expectations of high school students were questioned, and it was tried to determine how this situation changed during and after the COVID-19 pandemic. More importantly, it is aimed at determining which groups are more vulnerable in situations that may cause social isolation in future and offering solutions to them.

## 2 Materials and methods

### 2.1 Study design

The study was planned as a cross-sectional study. In the study, high school students or their relatives have experienced COVID-19, the change in their expectations for the future compared to the pre-COVID-19 pandemic, and the future expectation scale was applied by the face-to-face survey method. For the study, a questionnaire was applied to 412 high school students between 13 December 2021 and 01 April 2022. Participants were informed about the study through their teachers. The questionnaire form was administered to all participants by the same person.

### 2.2 Data collection tools

Participants were asked four questions about age, gender, and whether they or their relatives had COVID-19 infections. Researchers consisting of specialists in public health, pediatrics, and psychiatry scanned the literature and added four more questions similar to those questioned in studies on this subject. Although these questions are not on a standard scale, they were evaluated as supporting and confirming the Future Expectations Scale for Adolescents (FESA).

FESA developed by McWhirter and McWhirter in 2008 was used in the study to determine the future expectation levels of adolescents. FESA was translated into Turkish by Tuncer in 2011 (8, 10). The original scale consists of 25 items, and each question is scored between 1 and 7. When the scale questions were examined, the sub-dimensions in the scale were determined as work and education, marriage and family, religion and society, and health and life. The Cronbach’s alpha coefficient of these four different structures was found to be 0.925. In our study, this value was calculated as 0.923.

### 2.3 Statistical analysis

Analyses were evaluated in the 22.0 package program of SPSS (Statistical Package for Social Sciences; SPSS Inc., Chicago, IL). In the study, descriptive data were shown as *n* and % values in categorical data and mean ± standard deviation (mean ± SD) values in continuous data. Chi-square analysis (Pearson’s chi-square) was used to compare categorical variables between groups. The conformity of continuous variables to a normal distribution was evaluated by the Kolmogorov–Smirnov test. Mann–Whitney *U*-test was used to compare paired groups, and the Kruskal–Wallis test was used to compare more than two variables. The statistical significance level in the analyses was accepted as a value of *p* of <0.05.

## 3 Results

A total of 412 participants with a mean age of 15.3 ± 1.1 (min = 13; max = 18) were included in the study. A total of 60% of the participants are 15 years old and under, and 40% are over 15 years old. A total of 26.5% of the students had COVID-19, and the relatives of 83.7% had COVID-19. A total of 60.2% of the students were of the opinion that the pandemic would always continue. A total of 81.1% of the students stated that their education was disrupted due to the pandemic. A total of 45.4% of the students stated that the pandemic affected their choice of profession. A total of 63.6% of the students stated that they looked to the future with more hope before the pandemic. The scale sub-dimensions and total scores of the students are shown in Table 1.

**Table 1:** All characteristics of students.

		Number	%
Age, Mean ± SD		15.3 ± 1.1	
Age	≤15	247	60.0
	>15	165	40.0
Infected with COVID-19	Yes	109	26.5
	No	303	73.5
Relatives infected with COVID-19	Yes	345	83.7
	No	67	16.3
I believe that the pandemic will always continue	I agree	248	60.2
	I disagree	97	23.5
	Do not know	67	16.3
My education was interrupted due to the pandemic	I agree	334	81.1
	I disagree	64	15.5
	Do not know	14	3.4
The pandemic has affected my career choice	I agree	187	45.4
	I disagree	177	43.0
	Do not know	48	11.7
I was more hopeful for the future before the pandemic	I agree	262	63.6
	I disagree	108	26.2
	Do not know	42	10.2
FESA-work and education		4.9 ± 1.4	
FESA-marriage and family		4.0 ± 1.4	
FESA-religion and society		4.7 ± 1.8	
FESA-health and life		4.5 ± 1.5	
FESA-total		4.6 ± 1.2	

The work and education sub-dimension (*p* = 0.007), health and life (*p* = 0.001), and total score (p = 0.007) of those aged 15 and younger were found to be significantly higher than the scores of those aged more than 15 years. The scale sub-dimensions and total scores of those infected with COVID-19 were significantly lower than those who did not (*p* < 0.001). The scale sub-dimensions and the total score of those whose relatives were infected with COVID-19 were found to be significantly lower than the scores of those who were not (*p* < 0.05; Table 2).

**Table 2:** Comparison of scale scores by age and COVID-19 status.

		Work and education	Marriage and family	Religion and society	Health and life	Total
		Mean ± SD	Mean ± SD	Mean ± SD	Mean ± SD	Mean ± SD
Age	≤15	4.2 ± 1.9	3.5 ± 1.7	3.9 ± 2.1	4.0 ± 1.9	3.9 ± 1.6
	>15	3.6 ± 2.0	3.2 ± 1.7	3.5 ± 2.2	3.4 ± 1.8	3.4 ± 1.8
p^*^		**0.007**	0.077	0.082	**0.001**	**0.007**
Infected with COVID-19	Yes	2.5 ± 1.8	2.4 ± 1.7	2.5 ± 2.0	2.6 ± 1.8	2.5 ± 1.7
	No	4.5 ± 1.7	3.7 ± 1.6	4.2 ± 2.0	4.2 ± 1.7	4.2 ± 1.5
p^*^		**<0.001**	**<0.001**	**<0.001**	**<0.001**	**<0.001**
Relatives infected with COVID-19	Yes	3.8 ± 2.0	3.2 ± 1.7	3.7 ± 2.2	3.6 ± 1.9	3.6 ± 1.8
	No	4.7 ± 1.4	3.9 ± 1.4	4.3 ± 1.9	4.4 ± 1.6	4.4 ± 1.2
p^*^		**0.004**	**0.006**	**0.036**	**0.001**	**0.002**

^*^Mann–Whitney *U*-test was performed. Bold characters show statistical significance.

The scale sub-dimensions and total scores of those who thought that the pandemic will always continue, those who thought that their education has been disrupted due to the pandemic, those who thought that the pandemic has affected their choice of profession, and those who stated that they look to the future with more hope before the pandemic were found to be significantly lower (*p* < 0.05; Table 3).

**Table 3:** Comparison of the scale scores according to the thoughts about the pandemic.

		Work and education	Marriage and family	Religion and society	Health and life	Total
		Mean ± SD	Mean ± SD	Mean ± SD	Mean ± SD	Mean ± SD
I believe that the pandemic will always continue	I agree	3.7 ± 1.9^a^	3.1 ± 1.7^a^	3.6 ± 2.1^a^	3.5 ± 1.8^a^	3.5 ± 1.7
	I disagree	4.6 ± 1.9^b^	3.9 ± 1.7^b^	4.3 ± 2.2^b^	4.4 ± 1.9^b^	4.4 ± 1.7
	Do not know	4.1 ± 1.8^a,b^	3.3 ± 1.5^a,b^	3.6 ± 2.1^a,b^	3.8 ± 1.8^a,b^	3.7 ± 1.6
p^*^		**<0.001**	**0.002**	**0.005**	**<0.001**	**<0.001**
My education was interrupted due to the pandemic	I agree	3.8 ± 2.0^a^	3.2 ± 1.7^a^	3.6 ± 2.2^a^	3.6 ± 1.9^a^	3.6 ± 1.7^a^
	I disagree	4.7 ± 1.7^b^	4.1 ± 1.7^b^	4.5 ± 2.1^b^	4.4 ± 1.8^b^	4.5 ± 1.6^b^
	Do not know	4.2 ± 1.6^a,b^	3.4 ± 1.6^a,b^	3.8 ± 2.0^a,b^	3.9 ± 1.5^a,b^	3.9 ± 1.4
p^*^		**0.001**	**<0.001**	**0.005**	**0.007**	**<0.001**
The pandemic has affected my career choice	I agree	3.7 ± 2.0^a^	3.1 ± 1.7^a^	3.4 ± 2.2^a^	3.5 ± 2.0^a^	3.5 ± 1.8^a^
	I disagree	4.2 ± 1.9	3.6 ± 1.7	4.1 ± 2.1	4.0 ± 1.8	4.0 ± 1.6
	Do not know	3.9 ± 1.6^a,b^	3.5 ± 1.6^a,b^	4.1 ± 1.9^a,b^	3.8 ± 1.5^a,b^	3.8 ± 1.4^a,b^
p^*^		**0.032**	**0.017**	**0.004**	**0.04**	**0.014**
I was more hopeful for the future before the pandemic	I agree	3.7 ± 2.0^a^	3.2 ± 1.8^a^	3.6 ± 2.2^a^	3.6 ± 1.9^a^	3.5 ± 1.8^a^
	I disagree	4.5 ± 1.8^b^	3.7 ± 1.7^b^	4.3 ± 2.2^b^	4.1 ± 1.8^b^	4.2 ± 1.6^b^
	Do not know	4.2 ± 1.8^a,b^	3.4 ± 1.3^a,b^	3.7 ± 1.9^a,b^	3.8 ± 1.5^a,b^	3.8 ± 1.4^a,b^
p^*^		**0.001**	**0.04**	**0.011**	**0.039**	**0.004**

^*^Kruskal–Wallis test was performed. ^a,b^Group from which the difference originates. Bold characters show statistical significance.

It was observed that the score of those who were infected with COVID-19 thought that the pandemic would always continue (70.6%) was significantly higher than the score of those who did not (56.4%; *p* = 0.027). It was observed that the score of those who were infected with COVID-19 thought that their education was disrupted due to the pandemic (90.8%) was significantly higher than the score of those who did not (77.6%; *p* = 0.009). It was observed that the score of thinking that those who were infected with COVID-19 looked to the future with more hope before the pandemic (77.1%) was significantly higher than the score of those who did not (58.7%; *p* = 0.002; Table 4).

**Table 4:** Comparison of thoughts about the pandemic according to the situation of those previously infected with COVID-19.

		Infected with COVID-19		Did not infected with COVID-19		p^*^
		Number	%	Number	%	
I believe that the pandemic will always continue	I agree	77	70.6	171	56.4	**0.027**
	I disagree	17	15.6	80	26.4	
	Do not know	15	13.8	52	17.2	
My education was interrupted due to the pandemic	I agree	99	90.8	235	77.6	**0.009**
	I disagree	9	8.3	55	18.2	
	Do not know	1	0.9	13	4.3	
The pandemic has affected my career choice	I agree	55	50.5	132	43.6	0.455
	I disagree	43	39.4	134	44.2	
	Do not know	11	10.1	37	12.2	
I was more hopeful for the future before the pandemic	I agree	84	77.1	178	58.7	**0.002**
	I disagree	20	18.3	88	29.0	
	Do not know	5	4.6	37	12.2	

^*^Chi-square analysis was performed. Bold characters show statistical significance.

## 4 Discussion

Although the world experienced SARS, swine flu, bird flu in the 2000s, and the Hong Kong flu in 1968 before the COVID-19 pandemic, it has not encountered an epidemic as serious as the COVID-19 outbreak since the Spanish flu in 1914 (11). Therefore, the memory and experience of the generation experiencing the COVID-19 pandemic are insufficient to cope with the epidemic. Developing technology and scientific studies on this subject have enabled measures to be taken against the epidemic, the development of vaccines and drugs against the disease, and the work of the reflexes of the states. However, while doing all this, the psychosocial state and emotional and spiritual needs of society were pushed into the background. This has caused humanity, which had experienced its last serious pandemic in the Spanish flu in 1914, to have difficulties coping with and accepting this new reality.

In a study comparing the future expectations of adolescents between 2014 and 2021 in Norway, it was observed that the future expectations of young people changed negatively during the pandemic period. In the same study, no difference was observed according to the status of having a COVID-19 infection or closure period (12). In a study conducted with adolescents aged 15–17 in Antalya, Turkey, participants stated that they were generally worried about their future, but some participants thought that their job opportunities would increase due to the pandemic (13). In the study conducted in Italy in the early stages of the pandemic, most of the adolescents were worried about their short-term futures. A quarter of the participants stated that the decrease in their future expectation was due to the limitations caused by the closures (14). In a study conducted with school-age adolescents in South Korea in 2021, future expectations before and after the pandemic were compared, and a significant decrease was observed in future expectations after the pandemic (3). Our study was conducted with high school adolescents during the pandemic, and the future expectation scores of adolescents who were infected with COVID-19 and whose relatives were infected with COVID-19 were found to be significantly lower than those who did not. It can be assumed that the quarantine measures taken during the pandemic, hospitalization, and fear of death negatively affect future expectations.

The future expectations of those older than 15 years of age were significantly more affected than those younger than 15 years of age. The age of 15 can be defined as the age period in which the adolescent gets used to defining himself as an individual and the sense of responsibility brought about by being an individual begins to form. Therefore, it becomes very important to direct this age group to education and activities that will contribute to their development and future lives in closures and other extraordinary situations.

In the study conducted in South Korea, future life expectancy in the economic field was most affected due to the pandemic. In our study, future expectations in the marriage and family sub-categories were the most affected, while work and education were the least affected sub-categories (3). It is considered that this is a situation arising from the social structure. Namely, in Anatolian culture, while young people get married and start home, other sub-categories follow. In developed societies, on the other hand, since individualization and economic independence are prioritized, adolescents are more sensitive to negative experiences affecting their lives in categories that are more important to them. From this point of view, the necessity of considering regional differences in the measures to be taken during the pandemic period emerges. It should not be forgotten that the topics to be supported may change according to the priorities of the adolescents. Practical measures should be taken according to geographical region, socio-cultural structure, economic situation, and whether they are in urban or rural areas. It should not be forgotten that the subgroups that will be affected by the future expectations of adolescents, who are the most vulnerable group, may change, and regional support programs should be established accordingly.

It is certain that the pandemic has created negative experiences in adolescents as well as in all age groups. As a matter of fact, in our study, the future expectation scores of school-age adolescents who thought that their education was disrupted due to the pandemic and those who thought that the pandemic would never end were found to be significantly lower than those who did not.

In a study in Italy, a significant number of young people stated that their education was disrupted in the early stages of the pandemic and that they would stay indoors during the summer vacation. In our study, the scores of those who think that education is affected, those who think that the pandemic will never end, and those who have a more hopeful outlook on life before the pandemic were found to be significantly higher in those who were infected with COVID-19 compared to those who did not. During the epidemic, it is evaluated that adolescents are negatively affected both in their near-future expectations (school, social environment, etc.) and in their long-term future expectations (work, family, health, etc.).

This study has some limitations. The high age variability of the groups may create changes in future expectations. In addition, although data are collected from participants from more than one school, expanding the sample can provide more clear information about the findings.

## 5 Conclusion

Future expectations are a more sensitive concept in adolescents than in adults. Significant decreases were found in the scores of future expectations of those infected with COVID-19. Considering the effects of lockdowns on adolescents, school climates should be organized by considering the sensitivity of young people about their future expectations, and families and society should be made aware of this issue. Studies have determined that the scores of the sub-dimensions of future expectations vary by region, and this variability should also be considered while taking measures for this vulnerable group.

## Data availability statement

The raw data supporting the conclusions of this article will be made available by the authors, without undue reservation.

## Ethics statement

Ethics committee approval was obtained for the study from the Ethics Committee of Non-Interventional Clinical Researches of Adıyaman University with the decision dated 16 November 2021 and numbered 09-01. In order for the study to be carried out in schools, permission to apply the questionnaire was obtained from the Directorate of National Education with the approval of the District Governorship dated 07.12.2021 and numbered 38494692. The participants or their parents provided their written informed consent to participate in this study.

## Author contributions

MP and DE: conceptualization, design, and methodology. MP, EÖ, and YK: data collection. OK and YK: formal analysis, funding acquisition, and supervision. MP, DE, and EÖ: investigation and project administration. MP, EÖ, and OK: writing the original draft. YK, DE, EÖ, and OK: revising the manuscript. All authors contributed to the article and approved the submitted version.
