# Supplementary material for: Future expectation levels of adolescents during the COVID-19 pandemic
Source: Front Public Health. 2023 Nov 30;11:1199280. doi: 10.3389/fpubh.2023.1199280 (PMC10720309; doi:10.3389/fpubh.2023.1199280)
Supplement: Supplementary file 1 [file Data_Sheet_1.docx]

Questionnaire Form

1. Age
2. Gender 1. Male 2. Female
3. Have you ever had a COVID-19? 1. Yes 2. No
4. Have any of your relatives had COVID-19? 1. Yes 2. No
5. Please indicate whether you agree with the following statements

|  | I agree | I do not agree | I'm undecided |
| --- | --- | --- | --- |
| 1. I believe that the COVID-19 pandemic will always continue. |  |  |  |
| 2. My education was disrupted due to the COVID-19 pandemic. |  |  |  |
| 3. The COVID-19 pandemic affected my career choice. |  |  |  |
| 4. Before the COVID-19 pandemic, I was more hopeful for the future. |  |  |  |

1. Please indicate whether you agree with the following statements.

| When I'm an adult; | I absolutely do not believe. | I don't believe. | I partially believe. | I neither believe nor believe. | I partially believe. | I believe. | I firmly believe. |
| --- | --- | --- | --- | --- | --- | --- | --- |
| 1. I will have accomplished the things I want to achieve in my life. |  |  |  |  |  |  |  |
| 2. I can buy what I want. |  |  |  |  |  |  |  |
| 3. I will find a good job. |  |  |  |  |  |  |  |
| 4. I will reach my target level of education. |  |  |  |  |  |  |  |
| 5. I will find a job I enjoy. |  |  |  |  |  |  |  |
| 6. I will find a stable job. |  |  |  |  |  |  |  |
| 7. I will always make a living. |  |  |  |  |  |  |  |
| 8. I will feel safe. |  |  |  |  |  |  |  |
| 9. The money I earn will be enough for me and my life partner. |  |  |  |  |  |  |  |
| 10. My job will give me opportunities to be proud of myself. |  |  |  |  |  |  |  |
| 11. I will have a happy life. |  |  |  |  |  |  |  |
| 12. I will marry. |  |  |  |  |  |  |  |
| 13. I will have children. |  |  |  |  |  |  |  |
| 14. I will be married before the age of 25. |  |  |  |  |  |  |  |
| 15. My marriage will always last. |  |  |  |  |  |  |  |
| 16. My child will always live in peace. |  |  |  |  |  |  |  |
| 17. My child will have a long life. |  |  |  |  |  |  |  |
| 18. I will provide a safe environment for my child. |  |  |  |  |  |  |  |
| 19. I will regularly attend community and religious services. |  |  |  |  |  |  |  |
| 20. I will participate in religious activities. |  |  |  |  |  |  |  |
| 21. I will be a leader in my community. |  |  |  |  |  |  |  |
| 22. I will live a long life. |  |  |  |  |  |  |  |
| 23. I will eat a healthy diet. |  |  |  |  |  |  |  |
| 24. I will exercise. |  |  |  |  |  |  |  |
| 25. My health will be fine. |  |  |  |  |  |  |  |
